# Study on thermal health and its safety management mode for the working environment

**DOI:** 10.3389/fpubh.2023.1227630

**Published:** 2023-08-21

**Authors:** Jue Wang, Cheng Jiang, Guang Yang, Gang Bai, Shixuan Yu

**Affiliations:** ^1^Key Laboratory of Mine Thermodynamic Disasters and Control of Ministry of Education, Liaoning Technical University, Fuxin, Liaoning, China; ^2^School of Safety Science and Engineering, Liaoning Technical University, Fuxin, Liaoning, China; ^3^School of Civil Engineering, Liaoning Technical University, Fuxin, Liaoning, China

**Keywords:** thermal health, PDCA, safety management mode, labor protection, working environment

## Abstract

Thermal health concerns have gained significant attention due to the heightened health risks faced by workers who are exposed to extreme thermal environments for prolonged periods. To ensure the occupational health and safety of such workers, and to enhance work efficiency, it is imperative to examine the characteristics of thermal health in the working environment. This study proposes three key elements of thermal health in the working environment, namely thermal health states, absence of heat-related illnesses, and heat adaptability, which can be used to develop a safety management framework for thermal health. By exploring the interconnections between these elements, the study summarizes their features and outlines the necessary precautions to safeguard them. The PDCA (plan/do/check/action) cycle management mode is utilized as a framework, with the three components of thermal health forming the core, to establish a safety management mode for thermal health. To ensure that employees work in a safe, healthy, comfortable, and productive environment, the assessment and control objectives of the thermal environment are regularly revised through the use of labor protection technology and thermal environment control technology. This paper presents a PDCA cycle safety management mode based on the characteristics of thermal health, which offers novel insights and approaches for assessing and managing workers’ thermal health.

## 1. Introduction

Thermal health pertains to the influence of the thermal environment on human health and is a crucial component of occupational health, distinguished by its unique and significant impact compared to other aspects of occupational health (1). The effects of the thermal environment are ubiquitous in the production process, with workers continuously experiencing physiological and psychological changes due to thermal regulation. This not only affects their comfort and health but also their efficiency and product quality (2).

Heat exposure is a prevalent issue in various occupational settings, and the adverse effects of heat stress on workers are becoming increasingly alarming. Prolonged exposure to excessive heat may pose a threat to workers’ physiological health (3, 4). The high-temperature environment reduces the temperature differential between the body’s internal and external environments, leading to a state of heat stress. This results in difficulties in metabolic heat transfer and a significant increase in energy metabolism and oxygen consumption, with physiological consequences such as heart failure and hypoxia, as well as an increased likelihood of heat-related illnesses (5–7). Moreover, prolonged occupational heat stress is associated with various health issues in workers exposed to hot work environments (8). These health issues include an increased risk of developing and progressing renal/urologic anomalies (9), which could be attributed to the impact of heat stress on the body. Some research has suggested a possible role of heat-induced DNA damage in these health outcomes, though further investigation is required to establish a definitive link. Latzka and Montain (10) have shown that when the human body engages in physical work in a hot environment, excessive perspiration can result in abnormalities in water and salt metabolism, osmotic pressure imbalances, acid–base imbalances, and in severe cases, life-threatening cardiac dysfunction. Meshi et al. (11) conducted a cross-sectional study involving 60 miners working in extreme thermal environments and found that occupational setting in the mining area poses a significant risk of exposure to extreme heat conditions, which may contribute to the manifestation of symptoms related to heat-related illnesses. Sombatsawat et al. (12) conducted a semi-longitudinal study involving 22 male farm employees working outdoors in hot weather throughout a year of farming. The study results indicated that farmworkers experienced significant exposure to environmental heat stress, which was manifested in physical changes. Jafari et al. (13) investigated the association between heat stress exposure and immunological parameters among 55 foundry workers. The study demonstrated that under heat stress conditions, there may be a reduction in leukocyte levels and immunoglobulin concentration, potentially leading to the weakening and suppression of the human immune system. Workers in the steel industry who are exposed to high-temperature heat-stress environments face elevated risks of adverse heat-related health outcomes and an increased likelihood of developing heat-related illnesses (14).

The escalating incidence of heat-related mortality and morbidity has sparked an immediate and pressing need to address the health hazards posed by heat stress. As a result, numerous countries have intensified their endeavors in heat health prevention and research (15, 16). The Development of effective thermal health management strategies holds the potential to alleviate the consequences of environmental heat stress, thereby reducing the morbidity and mortality associated with heat-related illnesses (17, 18). Stephens et al. (19) conducted a comprehensive subjective thermal health survey to evaluate the well-being of workers exposed to thermal environments. Their study encompassed both qualitative and quantitative assessments, resulting in evidence-based thermal health strategies aimed at mitigating outdoor quarantine and reducing the risk of heat stress among the workforce. Smith et al. (20) examined the knowledge of heat-related first aid among 60 farm workers and concluded that training in first aid for heat-related symptoms could effectively prevent the occurrence of heat-related illnesses, ultimately reducing both morbidity and mortality rates. This study demonstrates the potential indirect mitigation of environmental heat stress through the implementation of training programs and effective management of thermal health-related knowledge. Specifically, providing first aid training enhances workers’ awareness of self-protection and equips them with the necessary knowledge to respond to heat-related illnesses, thereby reducing the duration of heat exposure and preventing conditions such as heat stroke (21). By recognizing the signs and symptoms of heat-related illnesses, workers can promptly administer first aid treatments, thus preventing further deterioration of these conditions among themselves and their colleagues, and ultimately reducing morbidity and mortality associated with occupational heat exposure. Notably, several countries have already implemented thermal health early warning systems (22). However, the absence of comprehensive thermal health safety management modes has raised concerns regarding the development of appropriate management approaches for various thermal environments (23). Addressing this concern is crucial for workers laboring in a thermal environment and necessitates swift action.

Prolonged exposure to high temperatures and thermal environments can induce substantial elevations in staff core temperature, heart rate, heat perception, and sweat loss, while concurrently a decreasing in physical mobility, cognitive function, and alertness, thereby impacting work efficiency and potentially contributing to safety incidents (24–28). O’Neal and Bishop (29) observed that individuals exhibited a marked increase in arithmetic errors, significantly reduced memory capacity, and notably delayed reaction times after working in a high-temperature environment. The working environment plays a pivotal role in influencing heat sensitivity, sustained work capacity, and acclimatization capability. Under certain circumstances, when ambient conditions deviate from a comfortable state, workers may experience reduced concentration and a decline in productivity (14). Liu (30) investigated the physiological and psychological hazards of coal miners in high-temperature and high-humidity environments, revealing that those working in such conditions exhibited significantly more physical, mental, and sensory symptoms compared to their counterparts in non-high-temperature settings. In extreme environments, the human body undergoes a range of physiological responses, including increased body temperature, elevated heart rate, fluctuations in blood pressure, and reduced concentration. Should specific physiological indicators exceed certain thresholds, this can lead to the development of heat-related illnesses, heightened labor risks, and even be hazardous to health (31).

Currently, thermal health research primarily focuses on understanding the mechanisms through which the thermal environment impacts human health However, a clear formulation of thermal health characteristics is yet to be established, leading to thermal health management being limited to passive management focused on avoiding occupational illness hazards. As the advancements in thermal environment control technology continue and labor protection technology, ensuring workers’ avoidance of occupational illness hazards will serve as the safety baseline for thermal health management. The ultimate goal of thermal health management will be to foster a thermally healthy and efficient working environment for workers. To achieve this, workers and managers must comprehend the effects of the thermal environment on the thermal health states and strengthen their awareness of labor protection ideologies. Taking proactive measures to manage workers’ thermal health states based on rational thermal health management objectives will be crucial.

This paper aims to consolidate current research findings on thermal health, delineate the characteristics of thermal health in the working environment, and summarize effective methods for ensuring thermal health to safeguard the occupational health and safety of workers in thermal environments. By establishing a safety management mode of thermal health in the working environment grounded in thermal health characteristics and integrating the PDCA (plan/do/check/action) cycle management mode, the paper seeks to enable workers to achieve optimal thermal health states in their work settings.

## 2. Thermal health in working environment

**Figure 1:**
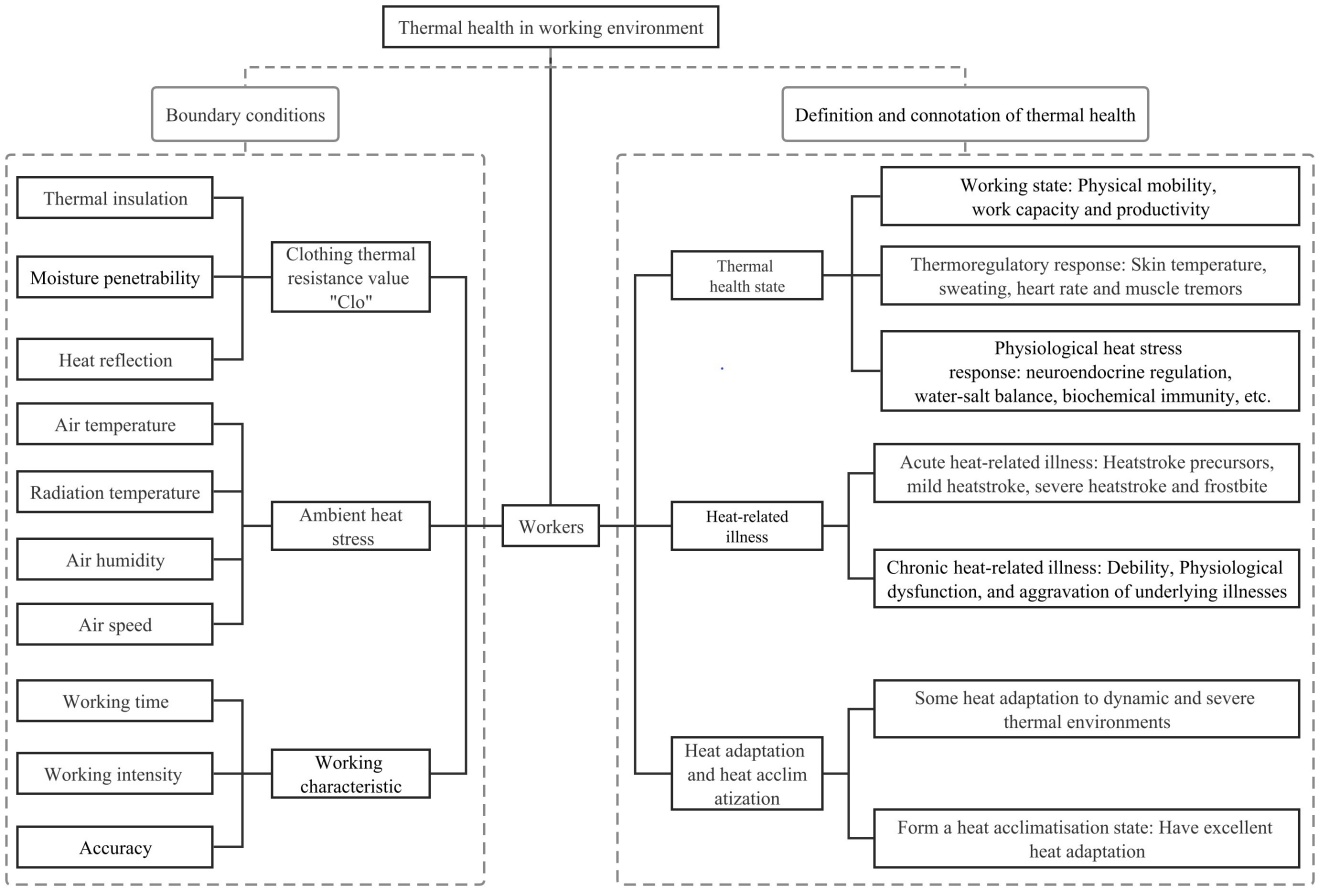
Diagram of thermal health in working environments.

Thermal health characteristics (Figure 1): A person achieves a thermal health state during work characterized by a high level of physical activity and work capacity, while considering other risk factors contributing to heat-related illnesses, such as medical conditions, medication usage, and predisposing risk factors. This state entails optimal physiological functioning of bodily systems, the absence of heat-related illnesses attributable to the physical thermal environment, effective heat adaptation to the dynamic thermal environment, and a certain level of tolerance to temperature extremes.

The thermal health characteristics encompass three essential components: Thermal health state, absence of heat-related illnesses, and heat adaptation. The thermal health state mirrors both the intensity of environmental heat stress and the physical condition of the worker (32). Heat-related illnesses result from prolonged exposure to excessive heat or extreme heat stress stimuli, and their definition involves considering physiological and psychological changes through a comprehensive assessment that integrates objective measurements with subjective perceptions (33). Heat adaptation, closely linked to the physical fitness and thermal experience of the worker (34), plays a crucial role in coping with thermal challenges. Thermal health refers to the body’s immediate response to ambient environmental heat stress, with its definition rooted in the interplay between the intensity of environmental heat stress and the body’s thermoregulatory abilities (35). A thermal health environment denotes a thermal environment where workers are maintained in a thermal health state (36).

Regarding the work process, a worker’s heat adaptation capacity depends on their pre-existing physical qualities, while the thermal health state manifests when the worker when encounters a thermal environment during work. Subsequently, heat-related illnesses may occur due to environmental heat stress experienced after work. Both heat adaptation and the absence of heat-related illnesses serve as physiological assurances for workers to attain thermal health states. Furthermore, a thermal health environment guarantees a thermal health state during work and the absence of heat-related illnesses after work.

A thermally healthy environment is crucial for promoting workers’ thermal health state, as the interaction between external heat stress and the body’s thermoregulatory mechanisms significantly influences thermal equilibrium. Understanding this interplay in compensable and uncompensable thermal environments is essential for comprehending thermal stress scenarios in various work settings. Thermoregulation depends on factors such as external environmental characteristics, individual metabolic rate, and heat transfer capacity to the skin. Physiological responses, such as blood vessel dilation, increased skin blood flow, and sweat evaporation, aid in maintaining temperature balance in high-temperature environments. However, individuals facing extreme heat conditions or with limited physiological capabilities may struggle to thermoregulate, necessitating appropriate measures to mitigate heat stress effects and ensure employee health and safety. By considering these factors, effective thermal health management strategies can prevent and alleviate health issues associated with high-temperature environments, ensuring workers maintain thermal balance in diverse heat stress scenarios.

In different working environments, the impact of heat stress can be categorized into two types: compensable and uncompensable (37). In compensable environments, the impact of heat stress can be effectively mitigated through environmental adjustments and appropriate measures, such as providing protective clothing, ventilation, cooling, and well-organized rest and hydration arrangements (38). Conversely, in uncompensable thermal environments, where extreme heat conditions cannot be easily modified solely through environmental adjustments, alternative measures become vital to alleviate the adverse effects of heat stress. These may include implementing reasonable work hours, job rotation, sufficient rest time, and conducting heat acclimatization training (21). Indeed, heat stress mitigation techniques, originally intended for compensable heat stress scenarios, can also be applied to cope with uncompensable heat stress situations. These measures are indispensable in both types of heat stress environments as they effectively alleviate the adverse effects of heat stress and ensure workers’ health. By employing these approaches, workers’ heat tolerance can be extended, and the rate of rise in body temperature can be reduced, providing significant relief in challenging heat stress conditions.

Moreover, the overall heat balance of the human body is influenced by the interplay between heat gain from the external environment and the reduction in metabolic heat production, as depicted in the boundary conditions in Figure 1. Ambient heat gain encompasses factors such as air temperature, radiation temperature, air humidity, and air velocity, while the decrease in metabolic heat generation is influenced by work intensity, work time, and work accuracy. The interplay of these factors directly impacts the thermal adaptation and health status of workers, particularly in various thermal scenarios.

In summary, an in-depth understanding of the distinctions between compensable and uncompensable thermal scenarios is paramount in developing effective thermal health management models. Proper environmental controls, personal protection measures, and work management strategies, can optimize workers’ health and comfort in uncompensable environments (20). Implementing suitable adaptive measures in uncompensable heat scenarios can reduce heat stress risks and safeguard workers’ physical and mental health (39). These practical insights and strategies serve as valuable references for thermal health management, ensuring workers can perform their duties safely and efficiently under diverse thermal conditions.

## 3. Analysis of the three elements of thermal health

### 3.1. Thermal health state

The thermal health state not only encompasses the physiological and psychological conditions of the human body under the influence of environmental heat stress but also significantly impacts worker productivity and product quality (40). Ensuring the thermal health of workers serves as a pivotal safeguard to prevent heat-related illnesses and promote both safety and efficiency in processes (1).

#### 3.1.1. Thermal regulation, heat stress, and thermal excitation

The body’s core temperature remains relatively constant, which is essential for supporting metabolism and vital activities (41). Achieving a physical thermal state equilibrium where heat production equals heat dissipation (42), plays a critical role in maintaining a stable core temperature. Heat production primarily depends on working intensity, while heat dissipation on the body’s surface is influenced by factors such as human skin temperature (43), skin moisture, clothing, air temperature in the work environment, radiation temperature, humidity, and wind speed (44). Human skin temperature and moisture act as physiological regulators, while environmental factors like ambient temperature, radiation, humidity, and wind speed can be artificially adjusted and serve as environmental heat stressors (38). Environmental heat stress factors can significantly impact the body’s thermoregulatory processes, disrupting the body’s thermal homeostasis and thermal health state (45). This disruption may lead to various heat-related illnesses, including heat exhaustion, heat cramps, and heat stroke. To maintain thermal equilibrium, the body transfers heat through dissipation from the body’s surface. If the amount of heat dissipated is insufficient to match the heat produced by metabolism, excess heat accumulation may raise core and skin temperatures (46), potentially leading to heat-related illnesses or a combination thereof.

Working in an extreme thermal environment necessitates the maintenance of thermal equilibrium through increased heat dissipation due to intense muscle activity generating substantial heat. However, the hot external environment hinders effective heat dissipation, resulting in a series of physiological heat stress reactions (32). These reactions primarily involve thermoregulation, water and salt metabolism, as well as changes in the cardiovascular, digestive, and neuroendocrine systems (47–49). Such changes represent the body’s compensatory responses to heat work within certain limits. However, if the heat load surpasses the body’s regulatory adaptation capacity, it can compromise the body’s thermal health.

Moreover, environmental thermal stresses can stimulate the skin with heat, causing additional thermal excitation during the work process. This increased thermal excitation may interfere with the work process and reduce worker productivity (50).

#### 3.1.2. Realization of the thermal health state

The fundamental prerequisites for achieving optimal thermal health are multifaceted. Firstly, the human body must be able to regulate its core temperature effectively, thereby ensuring a stable internal environment. Secondly, the dissipation of heat must be carried out in a compensatory manner, such that the level of compensation required does not pose any undue physiological or psychological strain on the individual. Furthermore, the thermal environment must maintain an appropriate level of thermal excitation, which facilitates efficient working conditions for the individual. By meeting these necessary conditions, one can achieve a state of thermal health that promotes optimal performance in the workplace.

Thermal health can be attained through job intensity grading and regulation, work time restriction control, thermal environment evaluation, air conditioning techniques, and cooling garments (39). The heat dissipation compensations used and the number of compensations consumed during thermal regulation of the body’s partitions can represent the state of thermal health (37). A thermal health state can be realized through thermal health management mode. At present, the standards and norms related to thermal health are stipulated in the occupational places, but there is no complete thermal health management mode, causing thermal health management to become passive management. To develop a reasonable and efficient thermal health management mode, it is necessary to refer to the relevant norms and standards of the industry and combine them with the PDCA cycle management mode to continuously improve the thermal health management objectives. To mitigate the impact of heat stress on workers when working in thermal environments, appropriate thermal health risk assessment strategies and standards have been developed and implemented. The Ergonomics of the Thermal Environment (DIN EN ISO 15265) (51) offers an essential framework to ensure worker health and safety. Hygienic Standards for the Design of Industrial Enterprises (GBZ 1-2010) (52) provides technical methods and control requirements for the prevention of heat and cold exposure in industrial settings with high and low-temperature environments. Public Health England has responded to heat events by devising and executing a series of thermal health indicators in response to heat events (53). Occupational Exposure Limits for Hazardous Agents in the Workplace Part 2: Physical Agents (GBZ2.2-2007) (54) sets workplace WBGT (wet bulb globe temperature index) limits based on different physical labor intensities and time exposure rates. The Classification of Occupational Hazards at Workplaces Part 3: Occupational Exposure to Heat Stress (GBZ/T 229.3-2010) (55) categorizes high-temperature work classes considering the duration of heat exposure and the thermal resistance of clothing. In The Ergonomics of the Thermal Environments (ISO 7730:2005) (56), methods are proposed to predict the degree of thermal sensation and discomfort in individuals exposed to thermal environments, delineating acceptable conditions for general thermal comfort and identifying instances of local discomfort. This aids in the assessment and determination of thermal health states. The Classification of Works in Cold Environment (GB/T 14440-93) (57) establishes criteria for quantifying the intensity of cold in work environments and its impact on human functions. World Health Organization (WHO) (58) has elaborated on the health risks of hot environments, offering methods to establish thermal health action plans and providing detailed disaggregated recommendations for preventing heat-related public health impacts. The Code for Design of Heating Ventilation and Air Conditioning in Industrial Buildings (GB 50019-2015) (59) sets clear objectives and techniques for indoor environmental control in industrial buildings, aiming to improve industrial enterprise labor conditions, enhance labor productivity, and ensure product quality and personal safety. The UKDH (UK Department of Health) (60) highlights in Local Government Leading for Public Health that the promotion and implementation of heat-related norms and standards by local governments can effectively enhance public health interventions.

### 3.2. Heat-related illnesses

Workers exposed to high-temperature environments experience environmental heat stress, which, in severe cases, can lead to lesions and physiological stress related to thermoregulation collectively known as heat-related illnesses due to their connection to heat stress (61). Occupational heat stress resulting from high-temperature thermal conditions significantly impacts the health, safety, and productivity of workers, and exceeding the limits of physical adaptation can result in heat-related illnesses. Kakamu et al. (62) conducted a study to investigate factors influencing the risk of heat-related illnesses in outdoor construction workers due to occupational heat stress. Their regression analysis revealed significant positive correlations with age, work area, maximum skin temperature, and post-warm-up heart rate, while construction experience showed a significant negative correlation. Another study by Nunfam et al. (63) focused on assessing occupational heat stress adaptation and social protection strategies for Ghana mining workers. They concluded that implementing adaptation policy options and recommendations aimed at overcoming barriers limiting workers’ and employers’ adaptive capacity had the potential to reduce workers’ vulnerability to occupational heat stress.

The high-temperature thermal environment exerts significant effects on various physiological functions of the body, including energy metabolism, water and salt metabolism, circulation, digestion, the neuroendocrine system, and the urinary system. Short-term heat stress can lead to acute heat-related illnesses such as heat stroke and frostbite, while prolonged exposure to high temperatures can result in chronic heat-related illnesses, characterized by physiological disorders, physical weakness, and exacerbation of underlying health conditions. Dukes-Dobos (64) classification categorizes chronic heat-related illnesses into three groups: category I—reduced thermal tolerance and diminished sweating capacity due to prior acute heat-related illnesses; category II—displaying less pronounced clinical symptoms, but similar to the general stress response; and category III—characterized by heat-related neurological weakness, anhidrosis, and an increased likelihood of kidney stones. Similarly, the cold environment can also impact human tissues, particularly through frostbite, frostnip, frozen stiffness, and skin damage when cold metals come into contact with the skin (65).

Heat-related illnesses have well-defined classifications in various norms and standards. In China’s Classification and Catalogue of Occupational Diseases (66), heat stroke resulting from high-temperature work settings is classified as an occupational illness attributed to physical factors. The Diagnosis of Occupational Heat Illness (GBZ 41-2019) (67) outlines diagnostic criteria and treatment guidelines for heat stroke aura, pyrexia, heat cramps, and heat exhaustion. Furthermore, the Technical Specification for Occupational Health Surveillance (GBZ 188-2014) (68) specifies the objective, substance, and frequency of occupational health checks before commencing work in high-temperature jobs, as well as during work and in emergencies. These classifications and standards play a vital role in raising workers’ awareness of heat-related illnesses caused by occupational heat stress. By incorporating different levels of occupational heat stress risk awareness and experience, along with appropriate industry norms and standards, workers can enhance their adaptation and resilience, taking proactive measures to manage heat stress and prevent heat-related illnesses effectively (23).

### 3.3. Heat adaptation and heat acclimatization

Heat adaptation refers to the body’s capacity to endure heat and encompasses a range of protective physiological responses to environmental heat stimuli, including behavioral, morphological, and genetic manifestations. The organism’s reflexive heat regulation gradually improves through repeated thermal stimuli, leading to the process known as heat acclimatization. Heat acclimation proves effective in reducing physiological stress during exercise-heat stress (69, 70). Workers who undergo heat acclimatization training demonstrate enhanced tolerance to heat stress in thermal environments, resulting in improved heat adaptation, such as reduced core temperature and heart rate, increased whole-body sweat rate, and extended duration of perceived exertion (71, 72). On the contrary, workers without heat acclimatization training experience lower heat adaptation and poorer thermoregulatory responses, leading to impaired work capacity (73) and an increased risk of heat-related illnesses, such as heat exhaustion or exertional heat stroke (74). In severe cases, insufficient heat adaptation capacity can lead to extreme hyperthermia and central nervous system complications, potentially causing multi-organ system failure and even death (75, 76). Hence, it is essential for workers in thermal environments to undergo heat acclimatization training. Studies have shown that combining hypoxia training with classical heat acclimatization training can more effectively promote the development of heat adaptation (77). Consequently, heat acclimatization training can significantly enhance workers’ heat adaptation in the same thermal environment. This improved heat adaptation results in a higher level of thermal comfort and heightened work efficiency for individuals who have undergone the training (78, 79).

The attributes of heat adaptation encompass both its establishment (speed and extent) and its potential decline. Achieving heat adaptation necessitates a specific time cycle, contingent on factors such as heat intensity, activity level, and the duration of heat acclimatization training. Typically, a standard heat acclimatization training program spans approximately 2 weeks to reach full development. However, it is important to note that de-acclimatization can occur if exercise is discontinued or when individuals leave the thermal environment, leading to a reduction in the previously acquired heat adaptation (80).

The Interpretation of Army Thermal Acclimatization Guide (81) aims to enhance the heat tolerance of soldiers and reduce the occurrence of heat stroke through heat acclimatization training. Ioannou et al. (82) provided a comprehensive summary of the current understanding regarding the impact of occupational heat stress on outdoor workers. They emphasized that ongoing anthropogenic global warming is expected to significantly affect physical work capacity, metabolic rate, and labor productivity potentially exceeding workers’ physical work capacity. In this context, heat acclimatization training emerges as a viable approach to enhance the heat adaptation of outdoor workers and improve their protection in challenging environments (83). Venugopal et al. (84) conducted a questionnaire survey involving 18 workers from 442 Indian workplaces to assess the impact of heat stress on health and productivity. Findings revealed that workers with high workloads experienced more heat-related health issues and lower productivity, especially among outdoor workers. Implementing heat acclimatization training could effectively improve workers’ heat adaptation, thus benefiting both the industry and the workers. The Classification of Occupational Hazards at Workplaces Part 3: Occupational Exposure to Heat Stress (GBZ/T 229.3-2010) (55) distinguishes the time limits or work between individuals who have undergone heat acclimatization and those who have not. Although heat acclimatization training can enhance heat adaptation (85), the extent of improvement varies significantly from person to person and cannot be simply differentiated by age, gender, fitness, or health states. Therefore, it cannot be considered an absolute guarantee against heat stroke (73).

## 4. Thermal health safety management mode

**Figure 2:**
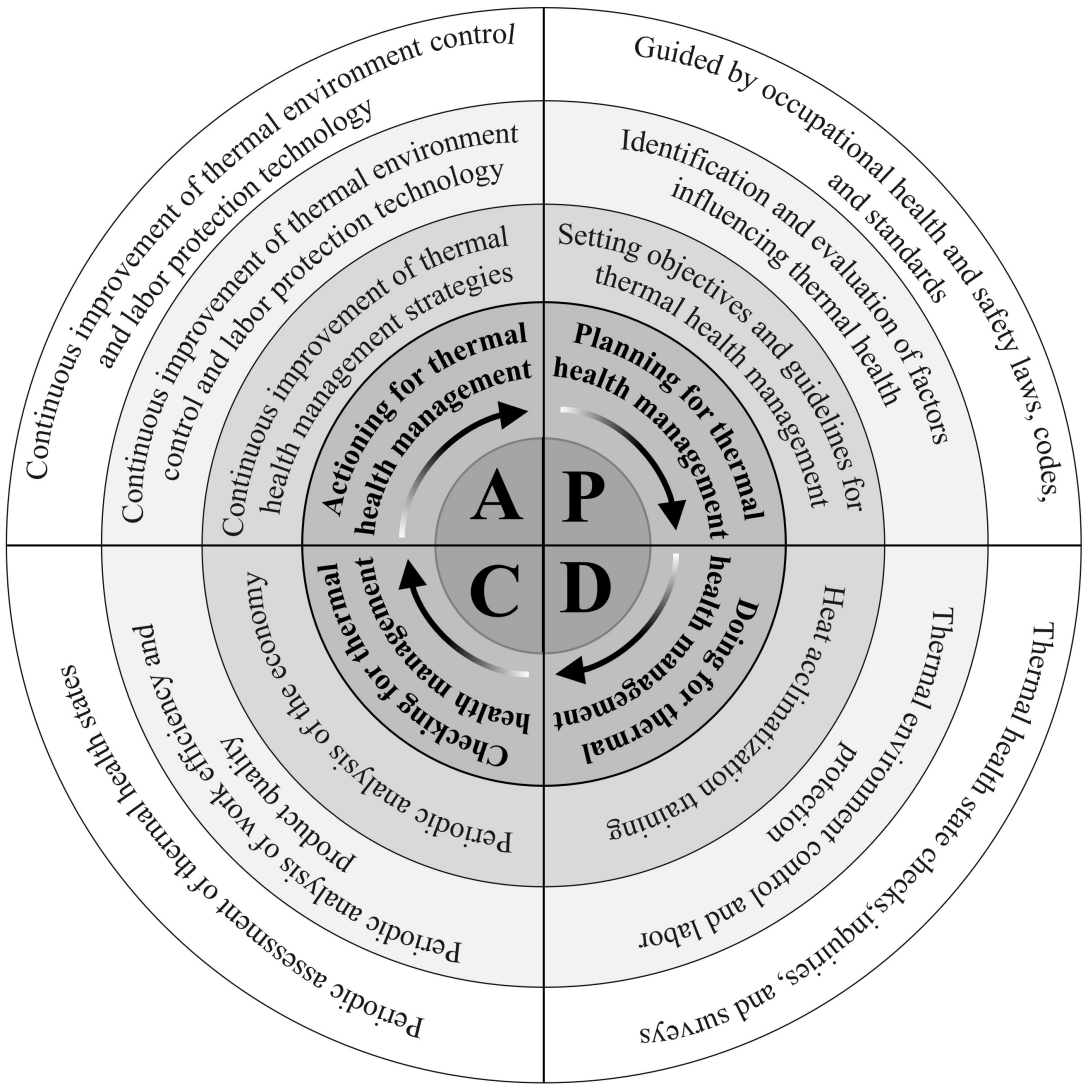
PDCA cycle safety management mode for thermal health in hot working environment.

The thermal health management mode operates on the foundation of the PDCA cycle management framework, with the three elements of thermal health at its core. It establishes a continuous cycle management process involving workers, the work environment, and the work process. This dynamic approach incorporates planning, doing, checking, and acting in a repetitive manner, facilitating constant refinement and enhancement of the management mode (Figure 2). This iterative thermal health cycle management strategy aims to achieve optimal outcomes in ensuring workers’ health and safety within thermal environments.

The thermal health safety management mode is not yet mature, requiring ongoing improvements to address diverse environmental parameters and workers’ physical indicators, thus enhancing management criteria. While certain countries have established thermal health guidelines, standards, and norms (86), their impact on managing various occupational thermal conditions remains limited (87). Although the PDCA cycle management mode has been effectively applied in thermal health warning systems (17), there is no complete PDCA thermal health management mode applied to occupational thermal settings since occupational thermal health is not clearly defined (23). Because the thermal environment is a physical environment system that can affect humans and their activities and is subject to change due to a variety of factors, the PDCA management mode’s continuous cycle of continuous improvement aids in the establishment of the thermal health safety management mode (88). In developing management standards and frameworks, valuable insights can be gained from PDCA systems or modes used to manage environmental, health, and safety aspects, ultimately enhancing the thermal health safety management mode (89, 90).

### 4.1. Planning for thermal health management (Plan)

Guided by occupational health and safety laws, codes, and standards: The foundation for thermal health management lies in adhering to occupational health and safety laws, norms, and standards (ISO 45001: 2018) (91). In particular industries such as the coal mining sector, thermal health management should be implemented according to industry-specific standards, like the Coal Mining Safety Regulations (92).

Identification and evaluation of factors influencing thermal health: The severity of heat stress in work environments is influenced by various factors, including ambient temperature, humidity, radiation, and wind speed. Additionally, work-related factors such as clothing intensity, duration, material fineness, and thermal resistance play a crucial role in thermal health management. To effectively manage heat stress, it is essential to detect and assess aspects like heat stress intensity and labor intensity in the thermal environment, using relevant codes and thermal health management guidelines as a basis for evaluation.

Setting objectives and guidelines for thermal health management: Thermal health management is anchored on the fundamental principle of safeguarding workers’ occupational health and safety. Its core objective is to enable workers to perform their tasks efficiently and effectively. To achieve this, cutting-edge thermal environment control technology and labor protection measures are harnessed to create a working environment that ensures workers’ safety, health, comfort, and optimal productivity.

### 4.2. Doing for thermal health management (Do)

Thermal health state checks, inquiries, and surveys: Workers undergo periodic medical examinations to assess their thermal health, focusing on detecting both acute and chronic heat-related illnesses resulting from heat stress in the workplace. In cases where individuals are diagnosed with heat-related illnesses, they are promptly removed from work for appropriate treatment and recovery. To further ensure workers’ health, a comprehensive thermal health management file is created for each employee. New workers or those returning from extended periods away from work undergo heat adaptation tests to ascertain their suitability for the specific job’s heat adaptation standards. If necessary, workers may be transferred to different positions to better accommodate their thermal health needs. To monitor individuals working in extreme heat environments, regular inquiries are made to promptly identify any signs of acute heat-related illnesses. Stricter work controls are implemented to regulate the frequency of work and rest periods for workers, ensuring adequate water intake to maintain their thermal health under challenging environments (93). These proactive measures enable managers to effectively manage and protect the thermal health of their workforce, promoting a safe and efficient working environment in thermal environments.

Thermal environment control and labor protection: Active regulation of ambient thermal parameters, using equipment like air conditioning, ventilation, and heating, is crucial for thermal health management. Workplace safety measures, such as limiting labor intensity and working hours, are essential to ensure worker safety under challenging thermal environments. Providing temperature-controlled equipment and salt supplements further safeguards workers, maintaining a comfortable environment and proper hydration. Employing these strategies effectively enhances thermal health and promotes worker safety and efficiency.

Heat acclimatization training: Heat acclimatization training is integrated into the pre-employment training for new workers or those returning to work after an extended period to enhance their heat adaptability and raise awareness of occupational heat stress (23).

### 4.3. Checking for thermal health management (Check)

Periodic assessment of thermal health states: During the production cycle, workers’ thermal health states are assessed against existing thermal health management strategies to evaluate the proportion of heat-related illnesses occurring during work. The relationship between workers’ thermal health levels and various influencing factors is analyzed to continuously improve thermal health management strategies, increase management effectiveness, and reduce the incidence of heat-related illnesses to ensure workers’ thermal health is maintained at a normal state.

Periodic analysis of work efficiency and product quality: To support adjustments to thermal health management objectives, the relationship between work efficiency, product quality, and workers’ thermal health states is analyzed within the context of an existing thermal health management strategy. This analysis provides valuable data that can be used to optimize thermal health management objectives.

Periodic analysis of the economy: The relationship between thermal environmental control, labor protection input, and production efficiency improvement is periodically analyzed to support the optimization of environmental control and labor protection strategies. In the management of thermal environments, economic analysis plays a crucial role. It enables the evaluation of the cost-effectiveness of thermal health management measures, facilitates the rational allocation of economic resources, and quantifies the impact of the thermal environment on labor productivity and overall enterprise economic performance.

### 4.4. Actioning for thermal health management (action)

Continuous improvement of thermal environmental control objectives: By systematically assessing the effects of thermal health management on workers’ physiological and psychological conditions, as well as their work efficiency and product quality, the goals for regulating the work environment are continuously revised and refined.

Continuous improvement of thermal environment control and labor protection technology: The perpetual enhancement of thermal environment control technology and labor protection measures is imperative to achieve thermal health management objectives, driven by an informed understanding of the worker’s thermal health states and guided by economic analysis. By consistently improving these management strategies, the aim is to optimize thermal health management, safeguard worker safety, and maximize overall productivity and efficiency in occupational thermal settings.

Continuous improvement of thermal health management strategies: The thermal health management mode comprehensive serves as the guiding framework for thermal health management. The intricate interplay of subjective and objective factors between the working environment, the working process, and the workers directly impacts thermal health conditions. To attain thermal health management objectives, companies must continuously refine and adapt specific thermal health management programs, ensuring their effectiveness in promoting worker health and productivity in thermal environments.

## 5. Conclusion

This study comprehensively summarizes workers’ thermal health characteristics in hot working environments to accurately determine their thermal health states. By examining the physiological state of workers in thermal conditions, including their working state, heat adaptation, and heat-related illnesses, we gain a holistic understanding of their health status, reflecting the impact of the hot environment on their well-being. The thermal health state directly indicates workers’ health and adaptation to heat stress while indirectly assessing ambient heat stress indicators. This serves as a foundational basis for establishing an effective occupational thermal health management system. The introduction of the three elements of thermal health, namely thermal health states, absence of thermal diseases, and thermal adaptation, elucidates the concept of thermal health in work settings, providing a fundamental framework for thermal health management mode.

Built upon the foundation of the three thermal health elements, we have adopted the PDCA cycle management mode is utilized as a framework to construct thermal health and safety management mode. With its emphasis on the three thermal health elements, this management approach facilitates a more direct evaluation of workers’ thermal health states. Through this comprehensive assessment, we aim to minimize the risk of heat stress in the thermal environment while providing intuitive management and safeguarding the health of workers. Integrating the three thermal health elements into the PDCA cycle management mode allows for the establishment of standardized thermal health management. By implementing management programs that encompass workers, the working environment, and the working process, we continuously cycle through the phases of planning, doing, checking, and actioning. This iterative approach leads to a constant enhancement of the thermal health management mode and strategy. As part of this dynamic process, we continuously update thermal environment evaluation parameters and control measures during the thermal health cycle management. By doing so, we refine thermal health management standards and employ advanced thermal environment control technology and labor protection measures. Through this management safeguard that workers are provided with a secure, healthy, comfortable, and productive working environment under various thermal conditions.

## Author contributions

JW and CJ conceived and drafted the manuscript. GY, GB, and SY revised the draft. All authors contributed to the article and approved the manuscript.

## Funding

This study was supported by National Outstanding Youth Science Fund Project of National Natural Science Foundation of China under grant no. 52104195.

## Conflict of interest

The authors declare that the research was conducted in the absence of any commercial or financial relationships that could be construed as a potential conflict of interest.

## Publisher’s note

All claims expressed in this article are solely those of the authors and do not necessarily represent those of their affiliated organizations, or those of the publisher, the editors and the reviewers. Any product that may be evaluated in this article, or claim that may be made by its manufacturer, is not guaranteed or endorsed by the publisher.
